# Implementation of Complementary Model using Optimal Combination of Hematological Parameters for Sepsis Screening in Patients with Fever

**DOI:** 10.1038/s41598-019-57107-1

**Published:** 2020-01-14

**Authors:** Jang-Sik Choi, Tung X. Trinh, Jihye Ha, Mi-Sook Yang, Yangsoon Lee, Yeoung-Eun Kim, Jungsoon Choi, Hyung-Gi Byun, Jaewoo Song, Tae-Hyun Yoon

**Affiliations:** 1 Center for Next Generation Cytometry, Hanyang University, Seoul, 04763 Republic of Korea; 2 Department of Chemistry, College of Natural Sciences, Hanyang University, Seoul, 04763 Republic of Korea; 3 Institute of Next Generation Material Design, Hanayng University, Seoul, 04763 Republic of Korea; 4 Department of Laboratory Medicine, College of Medicine, Yonsei University, Seoul, 03722 Republic of Korea; 5 Department of Laboratory Medicine, College of Medicine, Hanyang University, Seoul, 04763 Republic of Korea; 6 Department of Mathematics, College of Natural Sciences, Hanyang University, Seoul, 04763 Republic of Korea; 7 Division of Electronics, Information and Communication Engineering, Kangwon National University, Kangwon-Do, 25913 Republic of Korea

**Keywords:** Computational biology and bioinformatics, Data mining

## Abstract

The early detection and timely treatment are the most important factors for improving the outcome of patients with sepsis. Sepsis-related clinical score, such as SIRS, SOFA and LODS, were defined to identify patients with suspected infection and to predict severity and mortality. A few hematological parameters associated with organ dysfunction and infection were included in the score although various clinical pathology parameters (hematology, serum chemistry and plasma coagulation) in blood sample have been found to be associated with outcome in patients with sepsis. The investigation of the parameters facilitates the implementation of a complementary model for screening sepsis to existing sepsis clinical criteria and other laboratory signs. In this study, statistical analysis on the multiple clinical pathology parameters obtained from two groups, patients with sepsis and patients with fever, was performed and the complementary model was elaborated by stepwise parameter selection and machine learning. The complementary model showed statistically better performance (AUC 0.86 vs. 0.74–0.51) than models built up with specific hematology parameters involved in each existing sepsis-related clinical score. Our study presents the complementary model based on the optimal combination of hematological parameters for sepsis screening in patients with fever.

## Introduction

Sepsis, a dysregulated host response to infection, is a significant public health concern across the world, with more than >31 million cases annually and a mortality of 17%^1^. In the USA, the incidence rate of sepsis is 3 in 1000 individuals, which is 750,000 cases per year^2^. For each hour sepsis treatment is delayed, sepsis mortality increases by 7.6%^3^. Therefore, early intervention is important for patients with sepsis to increase the survival rates. However, the symptoms of sepsis are frequently non-specific, leading to a delay in diagnosis of sepsis. Fever, one of the symptoms, occurs in response to infection, inflammation and trauma. While it is often first manifestation of sepsis, it also is a sign of non-infectious etiology. Its non-specific response makes the early diagnosis of sepsis difficult. Given this difficulty with diagnosis, comparison study for two groups, patients with sepsis and patients with fever (abnormally high temperature), can therefore uncover set of (bio) markers which is sensitive and specific for sepsis screening in its early state.

Studies on biomarkers, clinical criteria, and predictive models have been conducted for sepsis diagnosis. Many biomarkers have been evaluated for their utility in sepsis diagnosis. It was previously confirmed that procalcitonin (PCT), lactate, C-reactive protein (CRP), cytokines, D-dimer, and pro-adrenomedullin are elevated in patients with systemic inflammation or bacterial infections^4–9^. The biomarkers related to hemocyte such as white blood cell (WBC) count, neutrophil, platelet count, bilirubin, and creatinine were reported^10–13^.

Among these biomarkers, PCT can be very high (>10 ng/ml) in sepsis and septic shock and has been used for diagnosis of bacterial sepsis and acts as a guide to discontinue antibiotic therapy. Therefore, PCT may be useful when evaluated in combination with patients’ overall condition and other clinical indexes due to its moderate diagnosis accuracy^14^. However, there are no data on routine procalcitonin level evaluation due to the absence of universal definition on how often PCT should be measured for diagnosis of sepsis and antibiotic treatment^15^. In addition, data on the PCT level are often missing because PCT testing is determined by the discretion of the physician. This limitation in available PCT data hampers its use for sepsis diagnosis modeling. Including PCT with many missing values to datasets may drastically reduce the number of observations available due to the missing-data pattern. Therefore, PCT was excluded in this study, while other hematological parameters, which are more often examined, were used to build up an auxiliary decision support system for sepsis screening in clinical practice.

Clinical score associated with infection and multiple organ dysfunction, such as SIRS, sepsis-related organ failure assessment (SOFA), quickSOFA (qSOFA) and logistic organ dysfunction system (LODS) were defined for sepsis diagnosis, prediction of severity and mortality^16–18^. The SIRS criteria are based on the presence of two abnormalities in heart rate, temperature, respiratory rate, and WBC count. Due to the low specificity of the SIRS criteria, a task force convened by national societies replaced the SIRS criteria with the SOFA in 2016. The SOFA score depends on a set of clinical parameters for quantifying organ dysfunction. The task force introduced the qSOFA which uses 3 clinical variables (respiratory rate, Glasgow Coma Scale score and systolic blood pressure), and has predictive validity outside of the ICU^19^. The LODS was compared with SOFA. Although the predictive capacity of SOFA and LODS were similar, the task force recommends using SOFA because it is better known and simper than LODS.

Predictive models using retrospective databases have been built to predict outcomes and prognosis of patients with sepsis and differentiate these patients from experimental cohorts^20–26^. In model development, logistic regression has mainly been used because the methodology is well established and coefficients can have intuitive clinical interpretations^27^. Some machine learning algorithms such as support vector machine (SVM) and random forest (RF) have been used. The models were developed using vital signs (e.g. blood pressure, heart rate, temperature, etc.), demographic data (e.g., birth weight, gestational age, gender, race, etc.), and clinical laboratory (e.g. C-reactive protein, procalcitonin, etc.) data with a few blood-related parameters, which have been considered as sepsis biomarkers (creatinine, WBC count, platelet count, and bilirubin).

The capacity of current predictive models on the early diagnosis of sepsis are listed in Table S1. The model performance varies with the experimental population, parameters and modeling algorithms, although all models show sensitivity over 0.5. Generally, there was a tradeoff between sensitivity and specificity. Most predictive models have been developed with multiple parameters shown in “blood test-related attribute” and “the other attributes” columns. Although the predictive models show good performance, increase in the number of parameters cause computational complexity of the models and make their interpretation difficult. Particularly, for the attributes related to vital signals and demographic information, measuring all the attributes is not efficient in the clinical situation and it also makes interpretation difficult. Additionally, the attributes available in each hospital may vary widely due to the absence of the required instrument. However, blood tests are widely used in most hospitals, since it is one of the most common types of medical tests. In our study, we have considered data availability and simplicity of model as key factors in identifying the optimal combination of hematological parameters for sepsis screening.

A few hematological parameters associated with organ dysfunction and infection are included in the biomarkers, clinical score and predictive models although various clinical pathology parameters (hematology, clinical chemistry and coagulation) in blood sample have been found to be associated with outcome in patients with sepsis. The investigation of the parameters facilitates the implementation of complementary model for screening sepsis.

In this study, statistical analysis on the multiple clinical pathology (hematological) parameters obtained from two groups, patients with sepsis and patients with fever, was performed and the complementary model was elaborated by stepwise parameter selection and machine learning. The predictive capacity of the developed complementary model was compared with models built with hematology parameters involved in each sepsis clinical score. Comprehensive data analysis by traditional statistic and machine learning approaches was performed to identify the optimal combination and to optimize the complementary model for sepsis screening.

## Materials and Methods

**Figure 1:**
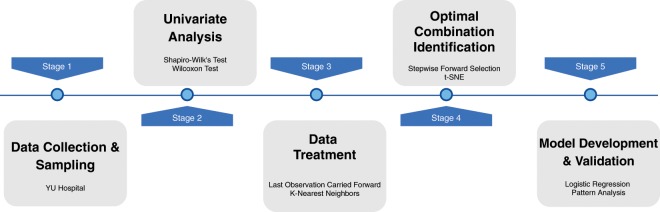
Overall workflow of this study.

Figure 1 shows the overall workflow of this study. From Yonsei University (YU) Severance Hospital in Rep. of Korea, retrospective dataset was collected.

In this study, the dataset was donated as YU-dataset (step 1). YU-dataset contains sepsis patients and control patients who showed a similar symptom with sepsis in terms of temperature. Univariate analysis was performed as a means of identifying the parameters with significant difference between sepsis and control group (step 2). The missing-data in the dataset was imputed by last observation carried forward (LOCF) and K-nearest neighbors (K-NN) before the optimal combination identification and model development and validation (step 3). The complete dataset was partitioned into training and validation set in a ratio of 7:3. The optimal combination was identified via model-based parameter importance measure and stepwise forward selection. In the stepwise forward selection, the optimal combination selection (step 4) and logistic regression-based model development (step 5) are performed in parallel. In step 4, t-SNE analysis for the optimal combination and each set of hematology parameters involved in SIRS, LODS and SOFA was carried to examine the underlying data patterns and trends of sepsis and control groups on the two-dimensional map. The model developed with the optimal combination was evaluated using the validation set in step 5. We additionally performed pattern analysis to discover beneficial patterns representing modality of clinical values of the optimal combination for sepsis screening.

### Data collection

Retrospective data (YU-dataset) from 7,743 patients (≥18 years) was collected from the clinical data management system (CDMS) of a Yonsei University Severance hospital in the Rep. of Korea from 2014 to 2017. The cohort consisted of 1,136 patients with sepsis and 6,607 control patients with high temperature (>38 °C). The retrospective data contains demographic (age, gender, and birthday), laboratory (chemistry, coagulation, and hematology blood test results) and clinical (date of diagnosis, date of the blood test, and ICD-10 code) data. The laboratory data contained 36 parameters as listed in Table S2: 15 serum-related parameters, 5 plasma-related parameters, and 16 whole blood-related parameters.

### Methods

#### Data sampling

Patients with sepsis were initially identified by the International Classification of Disease, 10th revision (ICD-10) code corresponding to sepsis. The sample of patients with sepsis was collected based on diagnosis date. If the laboratory data (a set of clinical values of the hematological parameters) existed on the date of diagnosis or within 7 days before the diagnosis date, the data was used as a sepsis sample. The laboratory data measured when a patient showed a symptom of high temperature (>38 °C) was used as control sample. The data obtained from YU Severance Hospital in Rep. of Korea was analyzed anonymously. Since (i) this research involves no more than minimal risk to the subject, (ii) the waiver of informed consent will not adversely affect the rights and welfare of the subjects and (iii) all data are anonymous, the informed consent was waived by the ethics committee/institutional review board of YU Severance Hospital (IRB no. 4-2017-1254). In addition, this study was approved by the institutional review board of YU Severance Hospital and all experiments were performed in accordance with relevant guidelines and regulations.

#### Univariate analysis

A normality test (Shapiro-Wilk’s Test) was performed to examine if the data of each parameter and group (sepsis and control) were normally distributed before univariate analysis, such as t-test and Wilcoxon test, to assess whether there was a statistically significant difference in a parameter between sepsis and control groups. When the normality assumptions were satisfied, a t-test was used. Otherwise, a Wilcoxon test was carried out. A parameter with a p-value smaller than 0.05 was considered to have a statistically significant difference with a 5% confidence level.

#### Data treatment

Missing data are unavoidable in clinical research^28–30^. Missing data can occur for multiple reasons: a loss to follow-up, failure to attend medical appointments, lack of measurements, failure to send or retrieve questionnaires, and inaccurate transfer of data from paper registration to an electronic database^31^. When large amounts of data are missing, the results of the subsequent analysis may be biased^32^. The missing values in the dataset were imputed via LOCF, which carries forward the last value that was present. The remaining missing values (less than 7%) were imputed by the K-NN method.

#### Identification of the optimal combination

The optimal combination was selected via stepwise-forward selection, which is a procedure that begins with an empty dataset and adds parameters to the regression or classification model one by one^33^. In each step of stepwise-forward selection, the most significant parameter is added to the dataset, and then the model performance is compared to identify the optimal combination. We estimated the importance of each parameter via model-based parameter importance measure, which is derived from the tuned model. To obtain the importance measure, a sepsis classification model for the training set was developed by logistic regression and then the absolute value of the regression coefficient divided by its standard error for each parameter was extracted. Afterward, the parameter with the highest absolute coefficient was added to the dataset in each step of stepwise-forward selection.

We additionally performed t-SNE analysis, a non-linear technique for reducing dimensionality that is particularly well suited for visualizing high-dimensional datasets^34^, on datasets for optimal combination and each set of hematology parameters involved in LODS and SOFA for visual interpretation of the underlying data patterns and trends of sepsis and control groups. Because only one hematology parameter (WBC count) is included in SIRS, t-SNE analysis for SIRS was not performed. To evaluate discriminatory ability of the set of hematology parameters in each score and the optimal combination identified in this study, Wilks’s lambda, a measure of how well each function separates cases into groups, was calculated. The parameters used for t-SNE analysis and Wilks’s lambda measurement are listed in Table S3.

#### Development and validation of complementary model

The YU-dataset was randomly divided into the training and validation dataset at a ratio of 70:30 for model development and validation. In each step of the stepwise forward selection, the same training and test label once divided was used for comparing performance under the same conditions. For model performance evaluation, confusion matrix and receiver operating characteristic (ROC) curve were used. The area under the curve (AUC) of ROC, sensitivity, specificity, positive predictive value (PPV), negative predictive value (NPV), and balanced accuracy were used as evaluation measures of the model. The sepsis group, a class of interest, is denoted as positive on the confusion matrix. To compare model performance in each step of stepwise forward selection, balanced accuracy, the average between sensitivity and specificity, was mainly used because the dataset was highly imbalanced. An optimal cutoff value (or optimal decision threshold) that maximizes the sum of sensitivity and specificity was selected in the model development. After the optimal combination selection, the performance of the complementary model was compared with models built with hematological parameters involved in each score, such as LODS, SOFA and SIRS. The parameters used for the comparison are listed in Table S3.

We additionally performed pattern analysis on the outcomes (true positive (TP), false positive (FP), false negative (FN) and true negative (TN)) resulted from validation phase to discover beneficial patterns representing modality of clinical values of the optimal combination for sepsis screening and to reveal limitation of our method using only hematological parameters in sepsis discrimination.

## Results and Discussion

### Data

**Figure 2:**
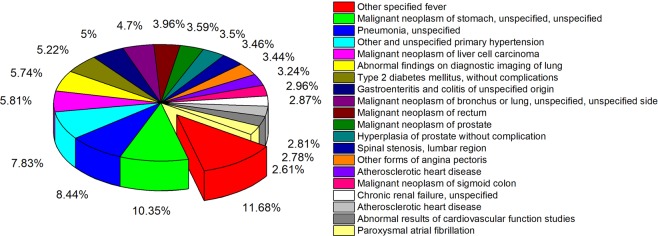
Proportion of top 20 diseases ranked based on frequency in control group.

Blood test results (7,743) for 1,136 patients with sepsis and 6,607 control patients from the YU-dataset were collected after data sampling. Figure 2 shows the proportion of top 20 diseases, such as malignant neoplasm, pneumonia, type 2 diabetes, chronic renal failure and heart diseases (Cardiovascular diseases such as hypertension, angina pectoris and atherosclerotic heart disease), ranked based on its frequency in control group.

Neoplastic diseases can cause fever^35^. The symptoms of pneumonia, an infection that inflames the air sacs in the lungs, include fever, chills and difficulty breathing. People with diabetes are more susceptible to developing infection because diabetes causes functional immune deficiency. Fever is one of warning signs to infection in patients with diabetes. Having kidney disease can weaken immune system, making it easier for infections. The patients with kidney disease are susceptible to various infections. Heart disease symptoms caused by heart infections include fever^36^. It is important to pay attention to patients with these diseases and fever to reduce risk of the development of sepsis. Sepsis is a potentially life-threatening condition caused by infection in blood and it induces multiple organ failure, leading cause of mortality. Therefore, screening sepsis from the other diseases with fever symptom is crucial for the appropriate treatment.

Sepsis can be difficult to diagnose because of non-specific symptoms, and the absence of fever in some cases. Figure S1 shows the proportion of the top 20 comorbidities ranked based on its frequency in the sepsis group. The comorbidities are important determinants of outcome in sepsis. Many patients with sepsis were identified as having comorbidities such as acute pyelonephritis, bronchitis, acute pharyngotonsillitis, pneumonia, acute renal failure and so on, which significantly contribute to sepsis development and could produce high temperature. Some comorbidities such as hypertension, constipation, atopic dermatitis, diabetes mellitus type 2, anemia and metabolic acidosis, which do not have a high temperature as the primary symptom, were included together. The heterogeneity in the comorbidities of sepsis patients makes the diagnosis of sepsis challenging. In this study, hematological parameters, which can help assess organ dysfunction and contribute to the clinical diagnosis and appropriate medical management of sepsis, were examined to assist in the diagnosis of sepsis and to suggest the optimal combination of the parameters more sensitive and specific for sepsis screening.

A common way to get data for classifier is to split the available data into two sets, a training set and validation set. Very often, the proportion chosen is 70% for the training set and 30% for the validation set. It was previously reported that the optimal proportion of cases for the training set tended to be in the range 40% to 80% and optimal model performance was obtained when 70% of the data was used for training and 30% of data was used for validation^37,38^. To build a well-performing classification model, it is essential that the training data has the same distribution as the validation data to which the model will be applied. In this study, Kolmogorov–Smirnov (KS) test was performed to test whether two samples, training and validation set, come from the same distribution. Because KS-test for 36 hematological parameters is not efficient, t-SNE was performed and then KS-test for two vectors on the 2D t-SNE map was carried out. The two vectors reduced in the 2D t-SNE map represent the high dimensional data. Figure S2 shows t-SNE plot where the blue cross and red circle indicate training data and validation data, respectively. Normal distribution curves of training data and validation data and p-value of KS-test are displayed on each axis of 2D t-SNE map. The 2D t-SNE plot, distribution curve and p-value indicate that the training data and validation set came from the same distribution.

### Univariate analysis results

The normality test results are listed in Table S4. Data were not normally distributed for most parameters except for PT (FIB) with p-value of 0.1844 in sepsis group. However, control data of PT (FIB) does not follow a normal distribution (p-value of 3.70E-24). Therefore, Wilcoxon tests were performed on all parameters. Table S5 shows the results of univariate analysis. In the table, the parameters are sorted in ascending order according to the p-value.

Three serum-related parameters (albumin, alkaline phosphatase (Alk# Phos), blood urea nitrogen (BUN)), one plasma-related parameter (PT (Prothrombin Time, Sec), and six whole blood-related parameters (Hemoglobin, mean platelet volume (MPV), red blood cell (RBC) Count, hematocrit (Hct.), red cell distribution width (RDW), and platelet (PLT) Count) were ranked in the top 10 based on the p-value.

Albumin and Alk# Phos showed the most significant differences. These are associated with sepsis-induced liver dysfunction^39^. Albumin levels are significantly altered in the critically ill. In the acute-phase response to trauma, inflammation, or sepsis, the level of albumin decreases^40^. Alk# Phos level is elevated in patients with sepsis^41^. BUN, one of the serum-related parameters identified to have a significant difference between groups, is elevated in patients with sepsis-induced kidney injury^42^.

The PT (sec) is prolonged in patients with sepsis^43^. The whole blood-related parameters, except for MPV and PLT count, are associated with RBCs. As sepsis is related to microcirculatory blood flow abnormalities leading to decreased RBCs deformability, impaired oxygen delivery to tissues, and organ failure, their levels are altered^44^. The MPV level is elevated in sepsis^45^. In sepsis, low PLT count is a well-known biomarker for severity^46^.

#### Identification of the optimal combination

Measuring all blood-related parameters is not efficient for diagnosing sepsis and is not always practical. Therefore, selecting important parameters is required. Table S6 lists the importance values of each parameter, which are derived from the tuned logistic regression model using the training set. In the table, the parameters are sorted in descending order according to their importance (absolute coefficient value of the tuned logistic model). The Pr (>|z|) column shows the two-tailed p-values testing the null hypothesis that the coefficient is equal to zero (i.e. no significant effect). Parameters with a two-tailed p-value of less than 0.05 were considered as significant variables. As shown in Table S6, the p-value tended to increase as the importance of each parameter decreased. To identify the optimal combination for sepsis screening, the parameters were added to model training and validation in the order shown in the table.

**Figure 3:**
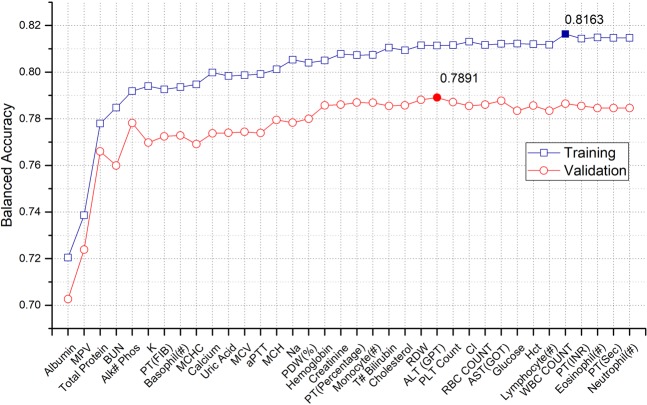
Model performance in each step of stepwise forward selection.

Figure 3 shows the model performance (balanced accuracy) in each step for the training and validation sets. The parameter sequentially added in each step to the dataset for model development is listed on the y-axis in Fig. 3. The numerical performance of the model on the training and validation phases are listed in Tables S7 and S8, respectively. Regarding model training, the model showed the highest balanced accuracy when 32 parameters were added to dataset. For the test, the model developed with 24 parameters showed the highest performance.

Regardless of the number of parameters used in model training and testing, the performance was not significantly improved after more than 17 parameters (up to hemoglobin) were used. In particular, the model performance sharply increased when parameters albumin, MPV, Total protein, BUN, and Alk# Phos were sequentially added to the datasets in both model training and validation. This indicates that the related parameters play key roles in sepsis discrimination, and a combination of parameters is more effective than any individual parameter. Consequently, albumin, MPV, Total protein, BUN, and Alk# Phos were involved in the optimal combination. Most of the parameters in the optimal combination except for MPV are variables of serum chemistry panel. This result is the natural consequences because level alternation of the serum-related variables of the combination is closely associated with organ (liver and kidney) dysfunction. On the other hand, it supports that use of the serum-related variables is effective to screen sepsis in patients with fever rather than hematology and coagulation parameters.

A visual exploration of the datasets for the optimal combination and sets of specific hematology parameters involved in each score was performed by t-SNE, which visualizes high-dimensional data by giving each data point a location in a two or three-dimensional map. The visual exploration, the core of exploratory data analysis, discovers meaningful information obscured by the complexity of data caused by its high dimensionality.

**Figure 4:**
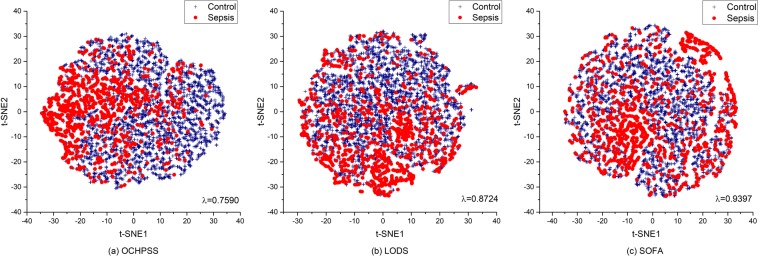
2D t-SNE plot for the optimal combination and sets of specific hematology parameters in each score.

For the LODS score, creatinine, total bilirubin, PLT count, WBC count, serum urea and prothrombin time (% of standard) were used in t-SNE analysis. In the case of SOFA score, creatinine, bilirubin and PLT count were used. Figure 4 shows 2D t-SNE plots for data sets comprised of the parameters of optimal combination and specific parameter in each score. The optimal combination is donated as “the optimal combination of hematological parameters for sepsis screening (OCHPSS)”. In Fig. 4, blue cross and red circle indicate the control and sepsis, respectively. As shown in Fig. 4, sepsis and control are relatively discriminated in the dataset comprised of the parameters of the optimal combination (a) better than the others (b and c). The measured Wilk’s Lambdas (λ) were 0.7590, 0.8724 and 0.9396 for the optimal combination, LODS and SOFA. Smaller λ value means greater discriminatory ability of the function. The t-SNE results and measured λ values indicate that the optimal combination identified are more sensitive and specific for sepsis than the sets of LODS and SOFA.

In this study, control samples were obtained from patients who already had disease and showed a symptom similar to patients with sepsis in terms of temperature. Therefore, the control and sepsis are not clearly distinguished on the t-SNE plot. This indicates that complete discrimination between them is difficult using the hematological parameters alone without other sepsis-specific biomarkers. In the case of sepsis samples, a dense cluster formed gradually from the right to left side on t-SNE plot of the optimal combination (Fig. 4(a)), revealing that the sepsis samples in the dense cluster have different patterns (or modalities) formed by the important hematological parameters compared to the other samples.

#### Development and validation of complementary model

**Figure 5:**
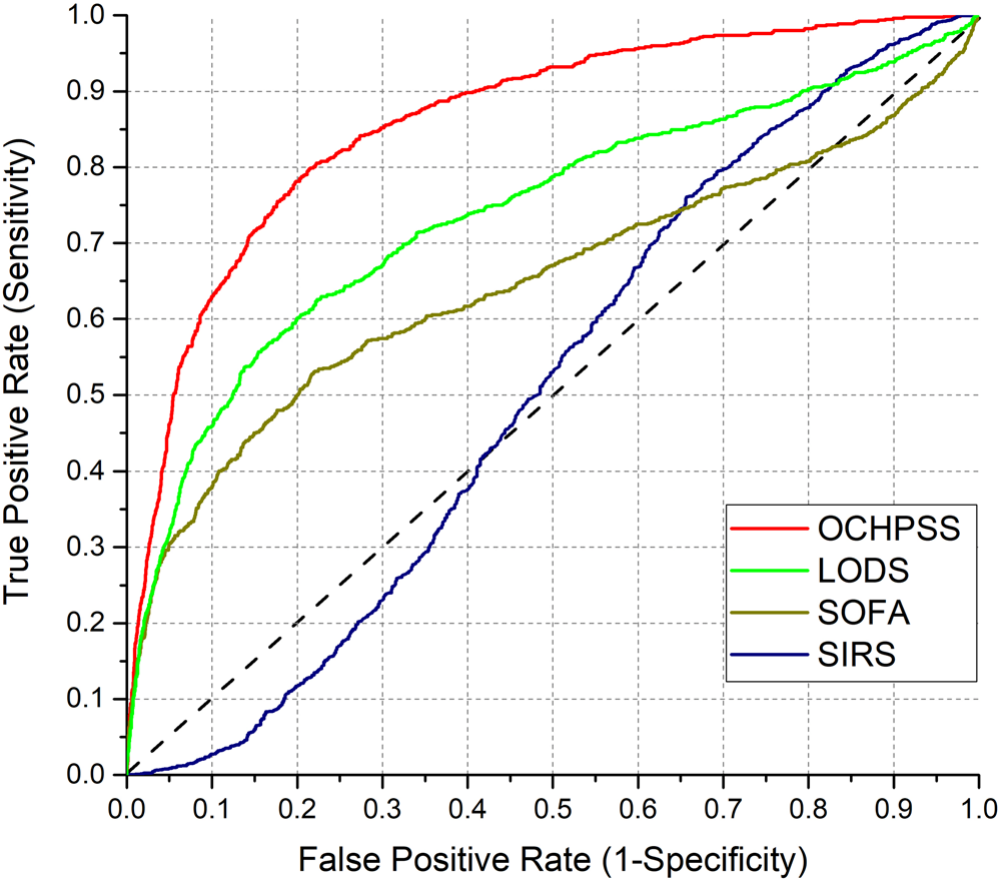
ROC curve for the optimal combination and sets of specific hematology parameters in each score.

Information of the developed complementary model using training dataset, such as coefficients, standard error, odds ratio, lower confidence interval (LCI), upper confidence interval (UCI) and *p*-value, are listed in Table S9. In Fig. 5, the ROC curves show the performance of models for the optimal combination and sets of specific hematology parameters in each score. The model developed with the optimal combination showed the best predictive capacity (AUC 0.8612) in sepsis discrimination in comparison with the other models. The model for LODS showed the second best result (AUC 0.7386). The model for SOFA provided relatively low performance (AUC 0.6487). While LODS and SOFA consist of same hematology parameters, such as bilirubin, creatinine and PLT count identified not to have significant difference between sepsis and control in the univariate analysis, the LODS additionally contains three more parameters, such as WBC count, serum urea associated with BUN and PT percentage. In this study, the BUN and PT percentage were identified to be sensitive and specific for sepsis screening. Therefore, the model for LODS provided better performance than SOFA. As shown in Fig. 5, the model for SIRS provided the lowest performance (AUC 0.5096). This result supports that the WBC count in SIRS is not sufficient to diagnosis sepsis and not an ideal marker in patients with fever.

The complementary model for the optimal combination provided the best predictive capacity in the validation as well as the training. The results for both training and validation datasets are listed in Table S10. The prevalence of preclinical disease (sepsis) is low, so even the training and validation with high sensitivity and specificity has a low PPV.

The common data organization strategies are crucial to discover insights from massive volumes of data. Because missing data are unavoidable in the clinical research, more sparsity of data occurs when moving to higher dimensions comprised of clinical target parameters. The sparsity of high dimensional data can significantly reduce available observations and cause an arbitrary bias in a model. To address the sparsity problem derived by high dimensional dataset, hematological parameters, which are more often examined, were selected and the missing values in the parameters were imputed by LOCF and K-NN approaches. The OCHPSS were determined from the processed data which consists of 7,743 observations and 36 parameters. Although the classification model using OCHPSS in this study shows the best performance, the predictive capability of OCHPSS and the generalization ability of the model should be validated before its practical use in order to prove that the dataset comprised of OCHPSS is enough to contain representative samples for the sepsis screening. For that, the prospective study and multicenter validation study should be carried out to validate the predictive capability of OCHPSS.

**Figure 6:**
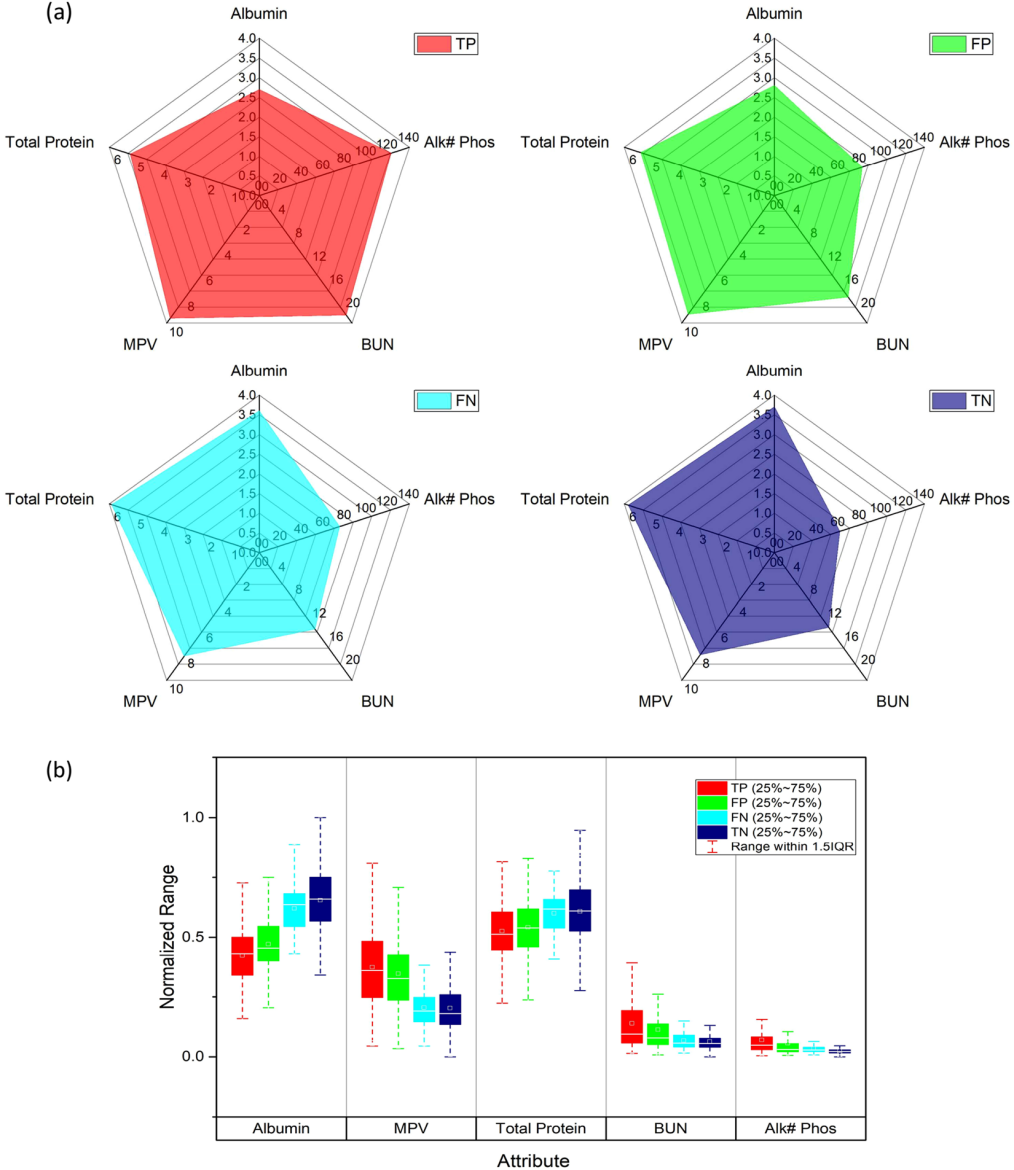
Radar pattern (**a**) and box plot (**b**) of clinical values of the optimal combination in the outcomes of the complementary model for the validation dataset.

Figure 6. shows radar pattern (a) and box plot (b) of clinical values of the optimal combination in each outcome (TP, FP, FN and TN) of the complementary model for the validation dataset. Table S11 lists descriptive statics for the outcomes. We expect that the range of clinical values of the parameters for TP in Table S11 would be complementary to existing sepsis-related clinical score. For radar patterns, the median value of each parameter was used. In Fig. 6(a), the radar plots show abundance of the median expression of each parameter among the outcomes. The expression level of Alk# phos, BUN and MPV in TP (sepsis) are higher than level expressed in the other cases, such as FP, FN and TN (control). In Fig. 6(b) box plot, the level of parameter-related key statistical properties decreases in the order of TP, FP, FN and TN. Unlike Alk# phos, BUN and MPV, the total protein and albumin in TP have lower expression level than expression in the other cases. The comparison of median expression level discovers that patients with sepsis have different hematological profiles compared to patients with fever. While a pattern modality of TP is similar to one of FP, the modality of TN (control) resemble one of FN. It indicates that the complete discrimination of sepsis using only hematological parameters is difficult. One the other hand, the false cases would be beneficial to estimate the likelihood of sepsis developing from patients with fever.

Most of the symptoms of sepsis are the same as in other conditions. It makes sepsis detection hard in its early stages. With this difficulty to diagnosis sepsis, underdiagnosis and overdiagnosis for cases in the decision border can be made^47^. It would contribute to increasing false negative and false positive cases. Thus, the complementary model or tool is needed to support the decision.

Sepsis is a major cause of death in hospitals. The risk of death from sepsis is as high as 30% from septic shock as high as 80%^48^. Early detection of sepsis with timely and appropriate interventions decrease mortality associated with septic shock^49^. Several predictive models for sepsis classification have been developed using multiple parameters obtained from related clinical tests to diagnose sepsis risk^20–26^. The multiple parameters were comprised of vital signals, blood test results, laboratory measurements, medical record text, demographic data, Glasgow coma score (GCS), and so on. Although those parameters help discriminate sepsis and predict sepsis risk, they increase the computational complexity of the model and make it difficult to interpret the prediction. In addition, measuring and integrating the many parameter values may be difficult in practice. In this study, we presented the complementary model developed with the optimal combination of hematological parameters identified to have sensitive and specific for sepsis screening. Merits of the hematological parameters are their relatively low cost, widespread applicability and short turnaround time. Therefore, we expect that the developed complementary model could be used as an auxiliary decision support system for sepsis screening in clinical practice.

The complementary model was developed using a dataset comprised of five hematological parameters. The frequent diseases in the control patient groups were “other specified fever”, “malignant neoplasm”, “pneumonia”, “type 2 diabetes”, “chronic renal failure” and “heart diseases”. Therefore, the model would be used as an auxiliary support system when sepsis is suspected from patients with those diseases. Defining the limitation of the model is crucial for the reliable application of the model. The definition of applicability domain (AD) of the model can help appropriate use of the complementary model. AD can be defined based on range-, distance-, probability- and density-based approaches^50^. The goal of applicability domain is to identify the region where the model’s predictions are reliable. The AD concept was derived from nano-informatics. With this concept, we defined range-based AD to limit use of the complementary model. Table S12 lists the range of the hematological parameters and age parameter from the dataset used for this study. We recommend using the complementary model with the consideration of the range of each parameter.

#### The complementary model and sepsis-3 definitions

New third international definitions for sepsis and septic shock were defined by a 2016 task force convened by the society of critical care medicine (SCCM) and the European society of intensive care medicine (ESICM)^19^. Sepsis (sepsis-3 definitions) is life-threatening organ dysfunction caused by dysregulated host response to infection. Organ dysfunction can be identified as an acute change in total sequential organ failure assessment (SOFA) score 2 points consequent to the infection. “Septic shock” is a subset of sepsis in which underlying circulatory, cellular and metabolic abnormalities are associated with a greater risk of mortality than sepsis alone. The task force noted that there are a number of novel biomarkers that can identify renal and hepatic dysfunction or coagulopathy earlier than the elements such as bilirubin and creatine used in SOFA, but these require broader validation before they can be incorporated into the clinical criteria describing sepsis. In this study, comprehensive data analysis on the 36 hematological parameters was performed and then the optimal combination of the parameters was identified. Although further validation of the predictive capability and reliability of the combination is needed, we expect that our findings would contribute to improving the SOFA in terms of hematological parameters. In our study, a specific control dataset was used to identify the combination and build the complementary model. It indicates that our finding was derived from limited data domain. Therefore, a study on the large dataset, which is enough to contain representative samples for the sepsis screening, should be performed.

## Conclusions

In this study, we investigated the hematological parameters to identify the optimal combination and developed the complementary model that would be used for sepsis screening in patients with fever. The developed model provided statistically better performance than models for the existing sepsis-related clinical score. We confirmed that the combination of the hematological parameters significantly contributes to increasing the sensitivity of our sepsis classification model. The hematological profiles for sepsis were presented via the pattern analysis in this study. The complete discrimination of sepsis was difficult because there are no parameters which have sufficient sensitivity and specificity for the sepsis classification. Thus, further study on harmonization of existing or new (bio) markers with the optimal combination of the hematological parameters for sepsis screening is needed to improve the predictive capacity of the developed complementary model.

## Supplementary information

Supporting information.

## Data Availability

The data that support the findings of this study are available from CNGC (Center for Next Generation Cytometry) but restrictions apply to the availability of these data, which were used under license for the current study, and so are not publicly available. Data are however available from the authors upon reasonable request and with permission of CNGC.
