# Supplementary material for: Implementation of Complementary Model using Optimal Combination of Hematological Parameters for Sepsis Screening in Patients with Fever
Source: Sci Rep. 2020 Jan 14;10:273. doi: 10.1038/s41598-019-57107-1 (PMC6959355; doi:10.1038/s41598-019-57107-1)
Supplement: Supplementary file 1 — Supporting information. [file 41598_2019_57107_MOESM1_ESM.docx]

**Implementation of Complementary Model
using Optimal Combination of Hematological Parameters
for Sepsis Screening in Patients with Fever**

Jang-Sik Choi^1,2^, Tung Xuan Trinh^1,2^, Jihye Ha^3^, Mi-Sook Yang^3^, Yangsoon Lee^4^, Yeoung-Eun Kim^4^, Jungsoon Choi^5^, Hyung-Gi Byun^6^, Jaewoo Song^3*^, and Tae-Hyun Yoon^1,2*^

^1^Center for Next Generation Cytometry, Hanyang University, Seoul 04763, Republic of Korea

^2^Department of Chemistry, College of Natural Sciences, Hanyang University, Seoul 04763, Republic of Korea

^3^Department of Laboratory Medicine, College of Medicine, Yonsei University, Seoul 03722, Republic of Korea

^4^Department of Laboratory Medicine, College of Medicine, Hanyang University, Seoul 04763, Republic of Korea

^5^Department of Mathematics, College of Natural Sciences, Hanyang University, Seoul 04763, Republic of Korea

^6^Division of Electronics, Information and Communication Engineering, Kangwon National University, Kangwon-Do 25913, Republic of Korea

*Corresponding Author:

*E-mail: taeyoon@hanyang.ac.kr (Tae Hyun Yoon)*

*E-mail: labdx@yuhs.ac (Jaewoo Song)*

Supporting information of this manuscript includes:
Supplementary Table S1. Previously Published Studies for sepsis classification

Supplementary Table S2. Parameters of the laboratory data

Supplementary Table S3. Parameters used for t-SNE and performance comparison of model

Supplementary Table S4. Normality test results (Shapiro-Wilk’s Test)

Supplementary Table S5. Univariate analysis results

Supplementary Table S6. Parameter importance derived from the tuned logistic regression model.

Supplementary Table S7. Model performance for training set in each step of stepwise forward selection

Supplementary Table S8. Model performance for validation set in each step of stepwise forward selection

Supplementary Table S9. Information of the complementary model for sepsis screening (cutoff: 0.1316)

Supplementary Table S10. Model Performance for training and validation set

Supplementary Table S11. Descriptive statics of each outcome and parameter for validation dataset

Supplementary Table S12. Range-based applicability domain of the complementary model

Supplementary Figure S1. Proportion of top 20 diseases ranked based on frequency in sepsis group

Supplementary Figure S2. 2D t-SNE map for training data and validation data

Supplementary Table S1. Previously Published Studies for sepsis classification

| **Ref. #** | **N positive** | **N negative** | **Population** | **Blood Test-related Attribute** | **The Other Attributes** | **Algorithm** | **Sensitivity** | **Specificity** | **PPV** | **NPV** | **AUC** |
| --- | --- | --- | --- | --- | --- | --- | --- | --- | --- | --- | --- |
| 20 | 521 | 2925 | Adults patients (> 18 years) in ICU (Intensive Care Unit) | BUN, Calcium, Creatinine, Glucose, Hematocrit, Hemoglobin, Magnesium, Phosphate, Platelet Count, WBC | Blood Pressure, Heart Rate, Respiratory Rate, Temperature, Anion Gap, Bicarbonate, | LR, SVM, LMT (logistic model tree) | 0.64 | 0.94 | 0.624 | 0.926 | 0.871 |
| 21 | 209 | 90 | Infants* in neonatal intensive care unit, late onset | Atyp Lymphs %, Baso (ABS), Basophils %, Bilirubin Conjugated, Calcium Ionized, Creatinine Blood, Eosinophil %, Glucose Whole Blood, Glucose Blood, Lymphs %, Lymphs (Abs), Atyp Lymphs (Abs), Metamyelocytes (Abs), Myelocytes (Abs), Metamyelo %, Mono (Abs), Monocytes %, Myelocytes %, Neutrophils %, Neutrophils (Abs), Platelet Count, Prothrombin Time INR, Patient Prothrombin Time, Bilirubin Total Blood, White Blood Cell Count | Ax Temp, Base Excess Arterial, Base Excess Venous, Base Excess Capillary , Bicarbonate , PCO2 Capillary , pH Capillary , CMB Temperature , C-Reactive Protein , CUM Transfusion , EO Automated Abs , Bicarbonate (Calc) , PCV Blood , Heart Plus Pulse , Heart Then Pulse , Heart Rate , Potassium Whole Blood , Lactate Whole Blood , Nt Automated Abs , Sodium Whole Blood , O2 Saturation , O2 Saturation (Calc), Parental Transfusion , Patient Partial Thromboplastin time , Pulse Rate , Respiratory Rate , Alt Blood , Serum Glutamic Oxaloacetic Transaminase or AST, Serum Glutamin Pyruvic Transaminase or ALT, Urine Glucose , Urine Leukocyte Esterase , Urine Nitrites , pCO2 Venous , pCO2 Arterial , Ph Arterial , Venous Ph , pO2 Arterial , PO2 Venous , Apgar Score (one minute) NICU, Apgar Score (five minutes) NICU, Apgar Score (ten minutes) NICU, Maternal Anesthesia NICU, Birthweight NICU, CRIBscore NICU, Chorioamnionitis NICU, Diabetes NICU, Resuscitation with Bag/Mask NICU, Resuscitation with Cardiac Comp. NICU, Resuscitation with Epinephrine NICU, Resuscitation with Intubation NICU, Resuscitation with Oxygen NICU, Substance Usage NICU, Fetal Monitoring NICU, Gestational Age (weeks) NICU, Gravida NICU, Ethniticity of the mother NICU, Length of the baby NICU, Meconium in Amniotic Fluid NICU, Mother's Age, Total babies in this pregnancy NICU, Mother's Race NICU, Preterm Labor NICU, Number of prev. deliveries NICU, Sex NICU, Vaginal Delivery NICU, Vaginal Presentation NICU, Vertex Presentation NICU, Is Sepsis Class Label | DT(CART), SVM, KNN, NBC, RF, LR(LibLinear), LBR, AODE, TAN | 0.88 | 0.18 | 0.71 | 0.381 | 0.61 |
|  |  |  |  |  |  |  |  |  |  |  |  |
| 22 | 7951 | 29875 | Infants | WBC count, Absolute neutrophil count (ANC), Immature-to-total neutrophil ratio (I/T ratio), and platelet count | sex, race, birth weight, gestational age (GA), inborn status, and Apgar score at 5 minutes | Logistic  regression | 0.55 | 0.95 | NA | NA | NA |
| 23 | 2164 | 163928 | Infants | WBC count, Absolute neutrophil count (ANC), Immature-to-total neutrophil ratio (I/T ratio), and platelet count | sex, race, birth weight, gestational age (GA), inborn status, and Apgar score at 5 minutes | Logistic  regression | 0.65 | 0.62 | NA | NA | NA |
| 24 | 2577 | 20276 | Intensive care unit (ICU) patients aged 15 years or more | - | systolic blood pressure, pulse pressure, heart rate, respiration rate, temperature, peripheral capillary oxygen saturation (SpO), age, and Glasgow Coma Score (GCS) | InSight, a machine learning classification system | 0.80 | 0.82 | NA | NA | 0.89 |
|  |  |  |  |  |  |  |  |  |  |  |  |
| 25 | 2297 | 23590 | Adult admitted to  ICU and non-ICU | Creatinine, Sodium, Chloride, Potassium, BUN, WBC, Hemoglobin, WBC Bands, Platelet count, Lactate, aPTT, Bilirubin, Glucose | temperature, diastolic blood pressure, systolic blood pressure, pulse oximetry, respiration rate, heart rate, Procalcitonin, INR, Microbiological cultures, NBRC, C-reactive protein, | Logistic  regression | 0.78 | 0.69 | NA | NA | NA |
|  |  |  |  |  |  |  |  |  |  |  |  |
|  |  |  |  |  |  |  |  |  |  |  |  |
| 26 | 32103 | 198833 | Patient admitted to emergency department | - | Age, gender, acuity, systolic blood pressure, diastolic blood pressure, heart rate, pain scale, respiratory rate, oxygen saturation, temperature, free text chief complaint, free text nursing assessment | linear SVM | 0.80 | 0.75 | 0.34 | NA | 0.85 |

Supplementary Table S2. Parameters of the laboratory data

| **No.** | **Whole Blood** | **Serum** | **Plasma** |
| --- | --- | --- | --- |
| 1 | MPV | Albumin | PT (FIB) |
| 2 | Basophil (#) | Total Protein | aPTT |
| 3 | MCHC | BUN | PT (Percentage) |
| 4 | MCV | Alk# Phos | PT (INR) |
| 5 | MCH | K | PT (Sec) |
| 6 | PDW (%) | Calcium |  |
| 7 | Hemoglobin | Uric Acid |  |
| 8 | Monocyte (#) | Na |  |
| 9 | RDW | Creatinine |  |
| 10 | PLT Count | T# Bilirubin |  |
| 11 | RBC COUNT | Cholesterol |  |
| 12 | Hct | ALT(GPT) |  |
| 13 | Lymphocyte (#) | Cl |  |
| 14 | WBC COUNT | AST(GOT) |  |
| 15 | Eosinophil (#) | Glucose |  |
| 16 | Neutrophil (#) |  |  |

Supplementary Table S3. Parameters used for t-SNE and performance comparison of model

| **No.** | **OCHPSS** | **SOFA** | **LODS** | **SIRS** |
| --- | --- | --- | --- | --- |
| 1 | Albumin | WBC Count | WBC Count | WBC Count |
| 2 | MPV | Total Bilirubin | Total Bilirubin |  |
| 3 | Total Protein | PLT Count | PLT Count |  |
| 4 | BUN |  | Serum Urea |  |
| 5 | Alk# Phos |  | Creatinine |  |
| 6 |  |  | PT (Percentage) |  |
| t-SNE | O | O | O | X |
| Model Validation | O | O | O | O |

Supplementary Table S4. Normality test results (Shapiro-Wilk’s Test)

| **No** | **Attribute** | **p-value** | | **No** | **Attribute** | **p-value** | | **No** | **Attribute** | **p-value** | |
| --- | --- | --- | --- | --- | --- | --- | --- | --- | --- | --- | --- |
|  |  | **Sepsis** | **Control** |  |  | **Sepsis** | **Control** |  |  | **Sepsis** | **Control** |
| 1 | Albumin | 8.86E-10 | 4.04E-19 | 13 | aPTT | 3.70E-24 | 3.70E-24 | 25 | Basophil (#) | 3.70E-24 | 3.70E-24 |
| 2 | Alk# Phos | 3.70E-24 | 3.70E-24 | 14 | Total Protein | 0.0046 | 3.48E-23 | 26 | Cl | 1.82E-14 | 3.70E-24 |
| 3 | Hemoglobin | 6.21E-13 | 0.0317 | 15 | Cholesterol | 6.36E-22 | 3.70E-24 | 27 | Monocyte (#) | 3.70E-24 | 3.70E-24 |
| 4 | MPV | 3.70E-24 | 3.70E-24 | 16 | PDW (%) | 8.87E-06 | 3.70E-24 | 28 | Eosinophil (#) | 3.70E-24 | 3.70E-24 |
| 5 | RBC COUNT | 1.56E-07 | 0.0117 | 17 | AST (GOT) | 3.70E-24 | 3.70E-24 | 29 | MCV | 2.18E-22 | 3.70E-24 |
| 6 | Hct | 1.82E-08 | 0.0006 | 18 | ALT (GPT) | 3.70E-24 | 3.70E-24 | 30 | PT(FIB) | 0.1844 | 3.70E-24 |
| 7 | RDW | 3.70E-24 | 3.70E-24 | 19 | Lymphocyte (#) | 3.70E-24 | 3.70E-24 | 31 | Na | 3.70E-24 | 3.70E-24 |
| 8 | BUN | 3.70E-24 | 3.70E-24 | 20 | Calcium | 1.38E-08 | 2.14E-21 | 32 | WBC COUNT | 3.70E-24 | 3.70E-24 |
| 9 | PT (Sec) | 3.70E-24 | 3.70E-24 | 21 | Creatinine | 3.70E-24 | 3.70E-24 | 33 | MCH | 3.70E-24 | 3.70E-24 |
| 10 | PLT Count | 3.70E-24 | 3.70E-24 | 22 | MCHC | 1.61E-05 | 3.70E-24 | 34 | Glucose | 3.70E-24 | 3.70E-24 |
| 11 | PT (Percentage) | 3.70E-24 | 3.70E-24 | 23 | T# Bilirubin | 3.70E-24 | 3.70E-24 | 35 | Uric Acid | 3.70E-24 | 3.70E-24 |
| 12 | PT (INR) | 3.70E-24 | 3.70E-24 | 24 | K | 3.70E-24 | 3.70E-24 | 36 | Neutrophil (#) | 3.70E-24 | 3.70E-24 |

Supplementary Table S5. Univariate analysis results

| **No.** | **Attribute** | **Sepsis** | | **Control** | | **p-value** |
| --- | --- | --- | --- | --- | --- | --- |
|  |  | **Mean (IQR)** | **SD** | **Mean (IQR)** | **SD** |  |
| 1 | Albumin | 2.83 (2.40- 3.20) | 0.63 | 3.49 (3.10-3.90) | 0.61 | 1.82E-204 |
| 2 | Alk# Phos | 164.19 (74.00-185.25) | 164.92 | 84.17 (50.00-87.00) | 88.55 | 3.54E-154 |
| 3 | Hemoglobin | 10.11 (8.80-11.30) | 1.87 | 11.70 (10.20-13.20) | 2.17 | 5.68E-147 |
| 4 | MPV | 9.50 (8.40-10.40) | 1.48 | 8.49 (7.70-9.10) | 1.13 | 8.99E-146 |
| 5 | RBC Count | 3.27 (2.82-3.68) | 0.64 | 3.78 (3.32-4.25) | 0.71 | 1.53E-138 |
| 6 | Hct. | 30.39 (26.40-34.00) | 5.58 | 34.76 (30.60-39.00) | 6.24 | 1.25E-133 |
| 7 | RDW | 15.66 (13.80-17.00) | 2.49 | 14.04 (12.80-14.60) | 2.05 | 2.42E-115 |
| 8 | BUN | 27.42 (12.80-34.03) | 22.95 | 16.96 (10.30-19.20) | 12.25 | 1.75E-79 |
| 9 | PT (Sec) | 15.92 (11.80-16.20) | 9.34 | 12.81 (11.10-13.03) | 4.80 | 7.93E-61 |
| 10 | PLT Count | 172.37 (75.00-236.25) | 126.09 | 207.20 (151.00-250.00) | 91.98 | 5.89E-58 |
| 11 | PT (Percentage) | 75.56 (61.00-96.00) | 23.35 | 87.53 (81.00-100.00) | 16.46 | 1.15E-53 |
| 12 | PT (INR) | 1.39 (1.03-1.41) | 0.84 | 1.12 (0.98-1.15) | 0.43 | 3.20E-53 |
| 13 | aPTT | 38.44 (29.80-40.00) | 18.32 | 32.44 (28.30-33.70) | 11.17 | 3.77E-43 |
| 14 | Total Protein | 5.69 (5.00-6.30) | 0.95 | 6.09 (5.40-6.80) | 0.93 | 7.03E-40 |
| 15 | Cholesterol | 128.36 (95.00-153.00) | 48.24 | 147.34 (119.00-173.00) | 43.04 | 7.71E-39 |
| 16 | PDW (%) | 55.28 (48.40-62.10) | 10.43 | 51.61 (46.20-56.30) | 7.83 | 3.30E-37 |
| 17 | AST (GOT) | 249.77 (20.00-67.00) | 1465.22 | 54.97 (18.00-38.00) | 187.47 | 1.35E-34 |
| 18 | ALT (GPT) | 92.25 (15.00-49.00) | 350.28 | 41.23 (12.00-31.00) | 107.26 | 4.21E-32 |
| 19 | Lymphocyte (#) | 0.95 (0.48-1.29) | 0.65 | 1.10 (0.67-1.40) | 0.65 | 1.35E-24 |
| 20 | Calcium | 8.28 (7.80-8.70) | 0.78 | 8.50 (8.10-8.90) | 0.67 | 1.02E-23 |
| 21 | Creatinine | 1.35 (0.62-1.45) | 1.43 | 0.99 (0.60-0.96) | 1.11 | 7.53E-22 |
| 22 | MCHC | 33.29 (32.50-34.10) | 1.31 | 33.57 (32.90-34.40) | 1.24 | 4.60E-21 |
| 23 | T. Bilirubin | 2.34 (0.50-1.90) | 4.4 | 0.99 (0.50-1.00) | 1.53 | 1.88E-19 |
| 24 | K | 4.00 (3.50-4.40) | 0.69 | 4.08 (3.80-4.40) | 0.51 | 9.84E-13 |
| 25 | Basophil (#) | 0.03 (0.01-0.04) | 0.07 | 0.02 (0.01-0.03) | 0.02 | 1.95E-06 |
| 26 | Cl | 100.82 (97.00-105.00) | 6.00 | 101.58 (99.00-104.00) | 4.64 | 2.26E-06 |
| 27 | Monocyte (#) | 0.50 (0.27-0.65) | 0.37 | 0.52 (0.33-0.65) | 0.28 | 1.11E-05 |
| 28 | Eosinophil (#) | 0.11 (0.02-0.14) | 0.15 | 0.11 (0.03-0.14) | 0.17 | 1.83E-05 |
| 29 | MCV | 93.37 (89.10-96.80) | 6.99 | 92.26 (89.00-95.50) | 6.06 | 2.61E-05 |
| 30 | PT (FIB) | 578.40 (427.50-729.25) | 222.70 | 555.26 (417.00-678.00) | 188.95 | 2.82E-05 |
| 31 | Na | 137.78 (134.00-141.00) | 5.99 | 138.01 (136.00-140.00) | 4.13 | 2.28E-03 |
| 32 | WBC COUNT | 10.50 (5.43-13.04) | 8.74 | 9.71 (6.68-11.89) | 5.97 | 3.02E-02 |
| 33 | MCH | 31.06 (29.70-32.30) | 2.39 | 30.99 (29.90-32.30) | 2.37 | 3.50E-02 |
| 34 | Glucose | 147.26 (102.00-170.00) | 71.47 | 136.62 (107.00-150.00) | 54.28 | 1.62E-01 |
| 35 | Uric Acid | 4.42 (2.60-5.60) | 2.51 | 4.25 (3.00-5.20) | 1.87 | 3.99E-01 |
| 36 | Neutrophil (#) | 8.87 (4.01-11.48) | 7.61 | 7.87 (5.02-9.94) | 4.29 | 4.15E-01 |

Supplementary Table S6. Parameter importance derived from the tuned logistic regression model.

| **No.** | **Parameter** | **Importance** | **Pr (>\|z\|)** |
| --- | --- | --- | --- |
| 1 | Albumin | 13.8134 | < 2e-16 |
| 2 | MPV | 9.6542 | < 2e-16 |
| 3 | Total Protein | 9.0682 | < 2e-16 |
| 4 | BUN | 6.2417 | 4.33E-10 |
| 5 | Alk# Phos | 5.9542 | 2.61E-09 |
| 6 | K | 5.4382 | 5.38E-08 |
| 7 | PT (FIB) | 4.7329 | 2.21E-06 |
| 8 | Basophil (#) | 4.5933 | 4.36E-06 |
| 9 | MCHC | 3.7854 | 0.0002 |
| 10 | Calcium | 3.6273 | 0.0003 |
| 11 | Uric Acid | 3.2609 | 0.0011 |
| 12 | MCV | 3.0383 | 0.0024 |
| 13 | aPTT | 2.9585 | 0.0031 |
| 14 | MCH | 2.8833 | 0.0039 |
| 15 | Na | 2.6197 | 0.0088 |
| 16 | PDW (%) | 2.5536 | 0.0107 |
| 17 | Hemoglobin | 2.5457 | 0.0109 |
| 18 | Creatinine | 2.4342 | 0.0149 |
| 19 | PT (Percentage) | 2.3846 | 0.0171 |
| 20 | Monocyte (#) | 2.3634 | 0.0181 |
| 21 | T# Bilirubin | 2.2479 | 0.0246 |
| 22 | Cholesterol | 2.0492 | 0.0404 |
| 23 | RDW | 1.9296 | 0.0537 |
| 24 | ALT (GPT) | 1.7479 | 0.0805 |
| 25 | PLT Count | 1.5246 | 0.1274 |
| 26 | Cl | 1.419 | 0.1559 |
| 27 | RBC COUNT | 1.2533 | 0.2101 |
| 28 | AST (GOT) | 1.2166 | 0.2238 |
| 29 | Glucose | 1.068 | 0.2855 |
| 30 | Hct | 0.9199 | 0.3576 |
| 31 | Lymphocyte (#) | 0.8851 | 0.3761 |
| 32 | WBC COUNT | 0.7385 | 0.4602 |
| 33 | PT(INR) | 0.7198 | 0.4716 |
| 34 | Eosinophil (#) | 0.2248 | 0.8221 |
| 35 | PT (Sec) | 0.0313 | 0.975 |
| 36 | Neutrophil (#) | 0.0163 | 0.987 |

Supplementary Table S7. Model performance for training set in each step of stepwise forward selection

| **Step** | **Parameter Added** | **TP** | **FN** | **FP** | **TN** | **Sensitivity** | **Specificity** | **PPV** | **NPV** | **BA** | **AUC** |
| --- | --- | --- | --- | --- | --- | --- | --- | --- | --- | --- | --- |
| 1 | Albumin | 533 | 263 | 1058 | 3567 | 0.6696 | 0.7712 | 0.3350 | 0.9313 | 0.7204 | 0.7870 |
| 2 | MPV | 549 | 247 | 983 | 3642 | 0.6897 | 0.7875 | 0.3584 | 0.9365 | 0.7386 | 0.8154 |
| 3 | Total Protein | 590 | 206 | 857 | 3768 | 0.7412 | 0.8147 | 0.4077 | 0.9482 | 0.7780 | 0.8446 |
| 4 | BUN | 611 | 185 | 916 | 3709 | 0.7676 | 0.8019 | 0.4001 | 0.9525 | 0.7848 | 0.8505 |
| 5 | Alk# Phos | 635 | 161 | 990 | 3635 | 0.7977 | 0.7859 | 0.3908 | 0.9576 | 0.7918 | 0.8612 |
| 6 | K | 656 | 140 | 1092 | 3533 | 0.8241 | 0.7639 | 0.3753 | 0.9619 | 0.7940 | 0.8643 |
| 7 | PT(FIB) | 653 | 143 | 1088 | 3537 | 0.8204 | 0.7648 | 0.3751 | 0.9611 | 0.7926 | 0.8644 |
| 8 | Basophil(#) | 647 | 149 | 1044 | 3581 | 0.8128 | 0.7743 | 0.3826 | 0.9601 | 0.7935 | 0.8662 |
| 9 | MCHC | 630 | 166 | 934 | 3691 | 0.7915 | 0.7981 | 0.4028 | 0.9570 | 0.7948 | 0.8659 |
| 10 | Calcium | 640 | 156 | 945 | 3680 | 0.8040 | 0.7957 | 0.4038 | 0.9593 | 0.7998 | 0.8689 |
| 11 | Uric Acid | 620 | 176 | 843 | 3782 | 0.7789 | 0.8177 | 0.4238 | 0.9555 | 0.7983 | 0.8682 |
| 12 | MCV | 628 | 168 | 886 | 3739 | 0.7889 | 0.8084 | 0.4148 | 0.9570 | 0.7987 | 0.8684 |
| 13 | aPTT | 638 | 158 | 940 | 3685 | 0.8015 | 0.7968 | 0.4043 | 0.9589 | 0.7991 | 0.8703 |
| 14 | MCH | 605 | 191 | 729 | 3896 | 0.7601 | 0.8424 | 0.4535 | 0.9533 | 0.8012 | 0.8718 |
| 15 | Na | 638 | 158 | 883 | 3742 | 0.8015 | 0.8091 | 0.4195 | 0.9595 | 0.8053 | 0.8727 |
| 16 | PDW(%) | 621 | 175 | 796 | 3829 | 0.7802 | 0.8279 | 0.4382 | 0.9563 | 0.8040 | 0.8737 |
| 17 | Hemoglobin | 631 | 165 | 845 | 3780 | 0.7927 | 0.8173 | 0.4275 | 0.9582 | 0.8050 | 0.8785 |
| 18 | Creatinine | 633 | 163 | 831 | 3794 | 0.7952 | 0.8203 | 0.4324 | 0.9588 | 0.8078 | 0.8786 |
| 19 | PT(Percentage) | 635 | 161 | 847 | 3778 | 0.7977 | 0.8169 | 0.4285 | 0.9591 | 0.8073 | 0.8791 |
| 20 | Monocyte(#) | 622 | 174 | 771 | 3854 | 0.7814 | 0.8333 | 0.4465 | 0.9568 | 0.8074 | 0.8818 |
| 21 | T# Bilirubin | 636 | 160 | 823 | 3802 | 0.7990 | 0.8221 | 0.4359 | 0.9596 | 0.8105 | 0.8824 |
| 22 | Cholesterol | 634 | 162 | 822 | 3803 | 0.7965 | 0.8223 | 0.4354 | 0.9591 | 0.8094 | 0.8819 |
| 23 | RDW | 627 | 169 | 762 | 3863 | 0.7877 | 0.8352 | 0.4514 | 0.9581 | 0.8115 | 0.8831 |
| 24 | ALT(GPT) | 653 | 143 | 914 | 3711 | 0.8204 | 0.8024 | 0.4167 | 0.9629 | 0.8114 | 0.8843 |
| 25 | PLT Count | 666 | 130 | 988 | 3637 | 0.8367 | 0.7864 | 0.4027 | 0.9655 | 0.8115 | 0.8844 |
| 26 | Cl | 640 | 156 | 823 | 3802 | 0.8040 | 0.8221 | 0.4375 | 0.9606 | 0.8130 | 0.8841 |
| 27 | RBC COUNT | 646 | 150 | 871 | 3754 | 0.8116 | 0.8117 | 0.4258 | 0.9616 | 0.8116 | 0.8848 |
| 28 | AST(GOT) | 652 | 144 | 901 | 3724 | 0.8191 | 0.8052 | 0.4198 | 0.9628 | 0.8121 | 0.8848 |
| 29 | Glucose | 670 | 126 | 1005 | 3620 | 0.8417 | 0.7827 | 0.4000 | 0.9664 | 0.8122 | 0.8851 |
| 30 | Hct | 669 | 127 | 1001 | 3624 | 0.8405 | 0.7836 | 0.4006 | 0.9661 | 0.8120 | 0.8851 |
| 31 | Lymphocyte(#) | 671 | 125 | 1015 | 3610 | 0.8430 | 0.7805 | 0.3980 | 0.9665 | 0.8118 | 0.8850 |
| 32 | WBC COUNT | 670 | 126 | 967 | 3658 | 0.8417 | 0.7909 | 0.4093 | 0.9667 | 0.8163 | 0.8858 |
| 33 | PT(INR) | 675 | 121 | 1014 | 3611 | 0.8480 | 0.7808 | 0.3996 | 0.9676 | 0.8144 | 0.8864 |
| 34 | Eosinophil(#) | 674 | 122 | 1004 | 3621 | 0.8467 | 0.7829 | 0.4017 | 0.9674 | 0.8148 | 0.8863 |
| 35 | PT(Sec) | 674 | 122 | 1005 | 3620 | 0.8467 | 0.7827 | 0.4014 | 0.9674 | 0.8147 | 0.8863 |
| 36 | Neutrophil(#) | 674 | 122 | 1005 | 3620 | 0.8467 | 0.7827 | 0.4014 | 0.9674 | 0.8147 | 0.8863 |

Supplementary Table S8. Model performance for validation set in each step of stepwise forward selection

| **Step** | **Parameter Added** | **TP** | **FN** | **FP** | **TN** | **Sensitivity** | **Specificity** | **PPV** | **NPV** | **BA** | **AUC** |
| --- | --- | --- | --- | --- | --- | --- | --- | --- | --- | --- | --- |
| 1 | Albumin | 216 | 124 | 456 | 1526 | 0.6353 | 0.7699 | 0.3214 | 0.9248 | 0.7026 | 0.8282 |
| 2 | MPV | 227 | 113 | 436 | 1546 | 0.6676 | 0.7800 | 0.3424 | 0.9319 | 0.7238 | 0.8300 |
| 3 | Total Protein | 250 | 90 | 403 | 1579 | 0.7353 | 0.7967 | 0.3828 | 0.9461 | 0.7660 | 0.8371 |
| 4 | BUN | 250 | 90 | 427 | 1555 | 0.7353 | 0.7846 | 0.3693 | 0.9453 | 0.7599 | 0.8526 |
| 5 | Alk# Phos | 265 | 75 | 442 | 1540 | 0.7794 | 0.7770 | 0.3748 | 0.9536 | 0.7782 | 0.8684 |
| 6 | K | 268 | 72 | 493 | 1489 | 0.7882 | 0.7513 | 0.3522 | 0.9539 | 0.7697 | 0.8816 |
| 7 | PT(FIB) | 270 | 70 | 494 | 1488 | 0.7941 | 0.7508 | 0.3534 | 0.9551 | 0.7724 | 0.8804 |
| 8 | Basophil(#) | 266 | 74 | 469 | 1513 | 0.7824 | 0.7634 | 0.3619 | 0.9534 | 0.7729 | 0.8764 |
| 9 | MCHC | 254 | 86 | 414 | 1568 | 0.7471 | 0.7911 | 0.3802 | 0.9480 | 0.7691 | 0.8651 |
| 10 | Calcium | 257 | 83 | 413 | 1569 | 0.7559 | 0.7916 | 0.3836 | 0.9498 | 0.7738 | 0.8670 |
| 11 | Uric Acid | 252 | 88 | 383 | 1599 | 0.7412 | 0.8068 | 0.3969 | 0.9478 | 0.7740 | 0.8553 |
| 12 | MCV | 255 | 85 | 399 | 1583 | 0.7500 | 0.7987 | 0.3899 | 0.9490 | 0.7743 | 0.8602 |
| 13 | aPTT | 260 | 80 | 430 | 1552 | 0.7647 | 0.7830 | 0.3768 | 0.9510 | 0.7739 | 0.8695 |
| 14 | MCH | 247 | 93 | 332 | 1650 | 0.7265 | 0.8325 | 0.4266 | 0.9466 | 0.7795 | 0.8393 |
| 15 | Na | 257 | 83 | 395 | 1587 | 0.7559 | 0.8007 | 0.3942 | 0.9503 | 0.7783 | 0.8625 |
| 16 | PDW(%) | 253 | 87 | 365 | 1617 | 0.7441 | 0.8158 | 0.4094 | 0.9489 | 0.7800 | 0.8522 |
| 17 | Hemoglobin | 260 | 80 | 383 | 1599 | 0.7647 | 0.8068 | 0.4044 | 0.9524 | 0.7857 | 0.8557 |
| 18 | Creatinine | 259 | 81 | 376 | 1606 | 0.7618 | 0.8103 | 0.4079 | 0.9520 | 0.7860 | 0.8551 |
| 19 | PT(Percentage) | 261 | 79 | 384 | 1598 | 0.7676 | 0.8063 | 0.4047 | 0.9529 | 0.7870 | 0.8582 |
| 20 | Monocyte(#) | 257 | 83 | 361 | 1621 | 0.7559 | 0.8179 | 0.4159 | 0.9513 | 0.7869 | 0.8479 |
| 21 | T# Bilirubin | 260 | 80 | 384 | 1598 | 0.7647 | 0.8063 | 0.4037 | 0.9523 | 0.7855 | 0.8579 |
| 22 | Cholesterol | 260 | 80 | 383 | 1599 | 0.7647 | 0.8068 | 0.4044 | 0.9524 | 0.7857 | 0.8583 |
| 23 | RDW | 257 | 83 | 356 | 1626 | 0.7559 | 0.8204 | 0.4192 | 0.9514 | 0.7881 | 0.8497 |
| 24 | ALT(GPT) | 270 | 70 | 428 | 1554 | 0.7941 | 0.7841 | 0.3868 | 0.9569 | 0.7891 | 0.8724 |
| 25 | PLT Count | 274 | 66 | 459 | 1523 | 0.8059 | 0.7684 | 0.3738 | 0.9585 | 0.7871 | 0.8804 |
| 26 | Cl | 260 | 80 | 384 | 1598 | 0.7647 | 0.8063 | 0.4037 | 0.9523 | 0.7855 | 0.8615 |
| 27 | RBC COUNT | 264 | 76 | 405 | 1577 | 0.7765 | 0.7957 | 0.3946 | 0.9540 | 0.7861 | 0.8670 |
| 28 | AST(GOT) | 267 | 73 | 416 | 1566 | 0.7853 | 0.7901 | 0.3909 | 0.9555 | 0.7877 | 0.8710 |
| 29 | Glucose | 273 | 67 | 468 | 1514 | 0.8029 | 0.7639 | 0.3684 | 0.9576 | 0.7834 | 0.8828 |
| 30 | Hct | 274 | 66 | 465 | 1517 | 0.8059 | 0.7654 | 0.3708 | 0.9583 | 0.7856 | 0.8827 |
| 31 | Lymphocyte(#) | 273 | 67 | 468 | 1514 | 0.8029 | 0.7639 | 0.3684 | 0.9576 | 0.7834 | 0.8833 |
| 32 | WBC COUNT | 272 | 68 | 450 | 1532 | 0.8000 | 0.7730 | 0.3767 | 0.9575 | 0.7865 | 0.8792 |
| 33 | PT(INR) | 277 | 63 | 483 | 1499 | 0.8147 | 0.7563 | 0.3645 | 0.9597 | 0.7855 | 0.8852 |
| 34 | Eosinophil(#) | 275 | 65 | 475 | 1507 | 0.8088 | 0.7603 | 0.3667 | 0.9587 | 0.7846 | 0.8835 |
| 35 | PT(Sec) | 275 | 65 | 475 | 1507 | 0.8088 | 0.7603 | 0.3667 | 0.9587 | 0.7846 | 0.8835 |
| 36 | Neutrophil(#) | 275 | 65 | 475 | 1507 | 0.8088 | 0.7603 | 0.3667 | 0.9587 | 0.7846 | 0.8835 |

Supplementary Table S9. Information of the complementary model for sepsis screening (cutoff: 0.1316)

| **No.** | **Variable** | **Coefficients estimated** | **Std. Error** | **Log (odds ratio)** | **LCI** | **UCI** | ***p-*value** |
| --- | --- | --- | --- | --- | --- | --- | --- |
| 1 | (Intercept) | -3.9894 | 0.4920 | -0.6387 | -0.8685 | -0.4108 | 4.46E-08 |
| 2 | Albumin | -2.0724 | 0.1127 | -3.9601 | -4.3861 | -3.5418 | 1.52E-75 |
| 3 | MPV | 0.4784 | 0.0370 | 2.3480 | 1.9934 | 2.7058 | 3.29E-38 |
| 4 | Total Protein | 0.6620 | 0.0711 | 2.4151 | 1.9093 | 2.9263 | 1.24E-20 |
| 5 | BUN | 0.0152 | 0.0026 | 1.3138 | 0.8711 | 1.7586 | 6.33E-09 |
| 6 | Alk# Phos | 0.0035 | 0.0004 | 3.6100 | 2.7551 | 4.4996 | 4.85E-16 |

Supplementary Table S10. Model Performance for training and validation set

| **Data Set** | **Method** | **TP** | **FN** | **FP** | **TN** | **Sensitivity** | **Specificity** | **PPV** | **NPV** | **BA** | **AUC** |
| --- | --- | --- | --- | --- | --- | --- | --- | --- | --- | --- | --- |
| Training | OCHPSS | 635 | 161 | 990 | 3635 | 0.7977 | 0.7859 | 0.3908 | 0.9576 | 0.7918 | 0.8612 |
|  | LODS | 497 | 299 | 1033 | 3592 | 0.6244 | 0.7766 | 0.3248 | 0.9232 | 0.7005 | 0.7386 |
|  | SOFA | 423 | 373 | 1020 | 3605 | 0.5314 | 0.7795 | 0.2931 | 0.9062 | 0.6554 | 0.6487 |
|  | SIRS | 523 | 273 | 3519 | 1106 | 0.6570 | 0.2391 | 0.1294 | 0.8020 | 0.4481 | 0.5096 |
| Validation | OCHPSS | 265 | 75 | 442 | 1540 | 0.7794 | 0.7770 | 0.3748 | 0.9536 | 0.7782 | 0.8353 |
|  | LODS | 190 | 150 | 451 | 1531 | 0.5588 | 0.7725 | 0.2964 | 0.9108 | 0.6656 | 0.6904 |
|  | SOFA | 172 | 168 | 442 | 1540 | 0.5059 | 0.7770 | 0.2801 | 0.9016 | 0.6414 | 0.6419 |
|  | SIRS | 223 | 117 | 1518 | 464 | 0.6559 | 0.2341 | 0.1281 | 0.7986 | 0.4450 | 0.5176 |

True Positive (TP), False Positive (FP), False Negative (FN), True Negative (TN), Positive Predictive Value (PPV), Negative Predictive Value (NPV), Balanced Accuracy (BA)

Supplementary Table S11. Descriptive statics of each outcome and parameter for validation dataset

| **Outcome** | **Parameter** | **Min.** | **1st Qu.** | **Median** | **Mean** | **3rd Qu.** | **Max.** | **SD** |
| --- | --- | --- | --- | --- | --- | --- | --- | --- |
| TP | Albumin | 1.1000 | 2.3000 | 2.7000 | 2.6586 | 3.0000 | 4.0000 | 0.4999 |
|  | MPV | 6.8000 | 8.6000 | 9.6000 | 9.7240 | 10.7000 | 14.8000 | 1.4277 |
|  | Total Protein | 2.2000 | 5.0000 | 5.5000 | 5.5924 | 6.2000 | 8.5000 | 0.9222 |
|  | BUN | 4.6000 | 13.4000 | 20.6000 | 29.5298 | 40.1000 | 201.0000 | 24.6060 |
|  | Alk# Phos | 19.0000 | 80.0000 | 123.0000 | 173.2498 | 204.0000 | 866.0000 | 151.8627 |
| FP | Albumin | 0.8000 | 2.5715 | 2.8017 | 2.8762 | 3.2000 | 4.2000 | 0.4915 |
|  | MPV | 6.7000 | 8.5000 | 9.3000 | 9.4778 | 10.2000 | 15.3000 | 1.3970 |
|  | Total Protein | 1.6000 | 5.1000 | 5.7000 | 5.7134 | 6.3000 | 9.2000 | 0.9595 |
|  | BUN | 3.4000 | 12.1250 | 17.5000 | 24.3291 | 28.9750 | 163.2000 | 20.0605 |
|  | Alk# Phos | 23.0000 | 58.0000 | 81.7985 | 142.4182 | 136.7500 | 2380.0000 | 203.6650 |
| FN | Albumin | 2.7000 | 3.2000 | 3.6000 | 3.5297 | 3.8000 | 4.7000 | 0.4357 |
|  | MPV | 6.8000 | 7.7000 | 8.1000 | 8.2173 | 8.6000 | 11.3000 | 0.8387 |
|  | Total Protein | 4.3000 | 5.7000 | 6.3000 | 6.1591 | 6.6000 | 7.5000 | 0.7047 |
|  | BUN | 5.0000 | 10.1000 | 13.2000 | 15.2427 | 19.6000 | 40.6000 | 7.2511 |
|  | Alk# Phos | 27.0000 | 61.0000 | 75.0000 | 89.1391 | 101.0000 | 236.0000 | 43.6495 |
| TN | Albumin | 1.8000 | 3.3000 | 3.7000 | 3.6821 | 4.1000 | 5.2000 | 0.5211 |
|  | MPV | 6.4000 | 7.6000 | 8.0000 | 8.2063 | 8.7000 | 11.8000 | 0.8786 |
|  | Total Protein | 2.9000 | 5.6000 | 6.2345 | 6.2188 | 6.9000 | 8.8000 | 0.8853 |
|  | BUN | 1.8000 | 9.8000 | 12.9000 | 14.4680 | 17.1000 | 79.0000 | 7.4833 |
|  | Alk# Phos | 8.0000 | 48.0000 | 61.0000 | 67.4017 | 76.0000 | 497.0000 | 33.1551 |

Supplementary Table S12. Range-based applicability domain of the complementary model

| **Parameter** | **Albumin** | **MPV** | **Total Protein** | **BUN** | **Alk# Phos** | **Age** |
| --- | --- | --- | --- | --- | --- | --- |
| Min | 0.80 | 6.40 | 1.60 | 1.80 | 8.00 | 18.00 |
| Max | 5.20 | 17.70 | 10.00 | 201.00 | 2380.00 | 96.00 |
| Average | 3.41 | 8.59 | 6.03 | 18.26 | 93.47 | 65.02 |
| SD | 0.65 | 1.21 | 0.93 | 14.61 | 100.91 | 14.95 |

**Supplementary Figure S1. Proportion of top 20 diseases ranked based on frequency in sepsis group**


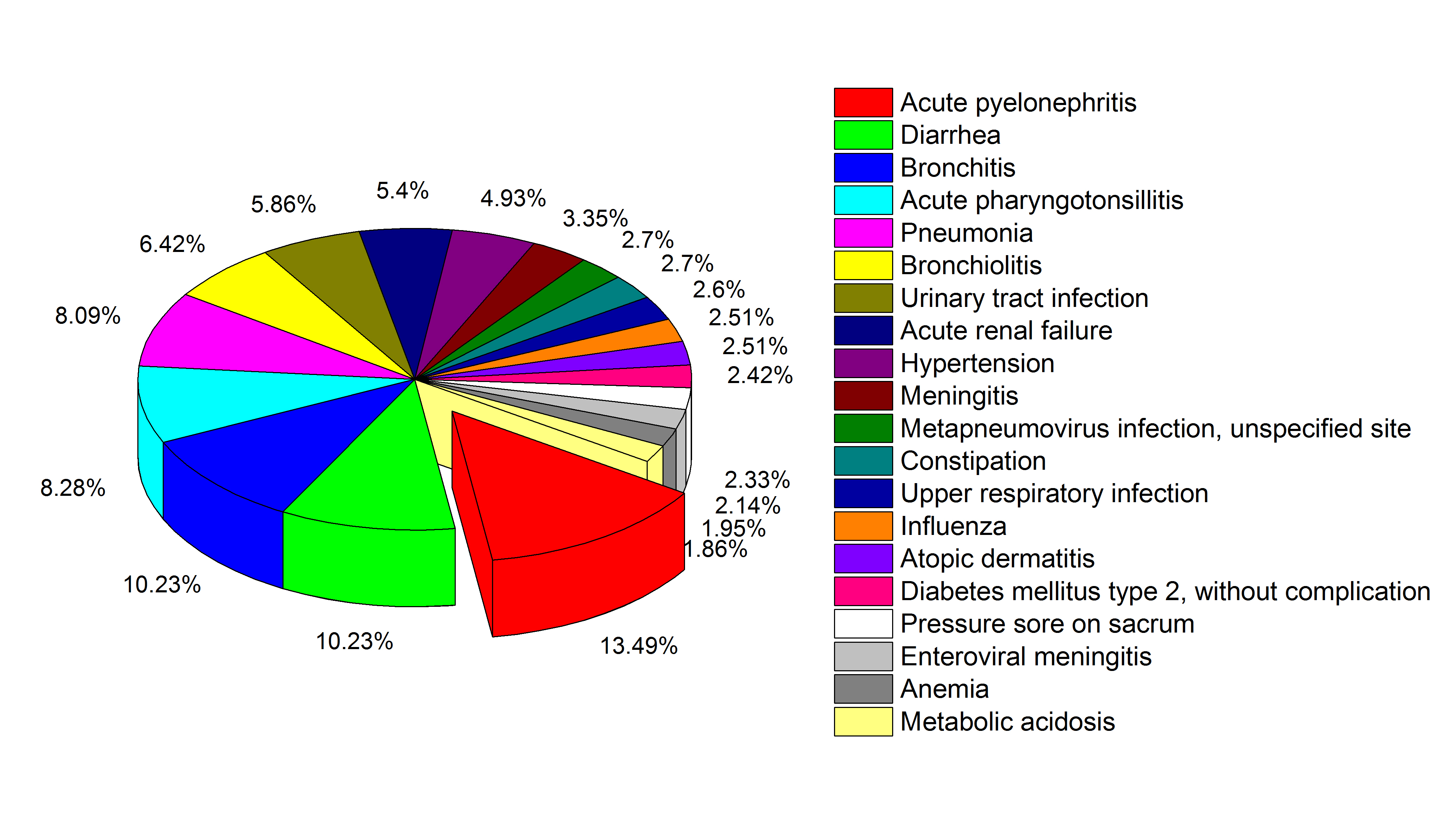


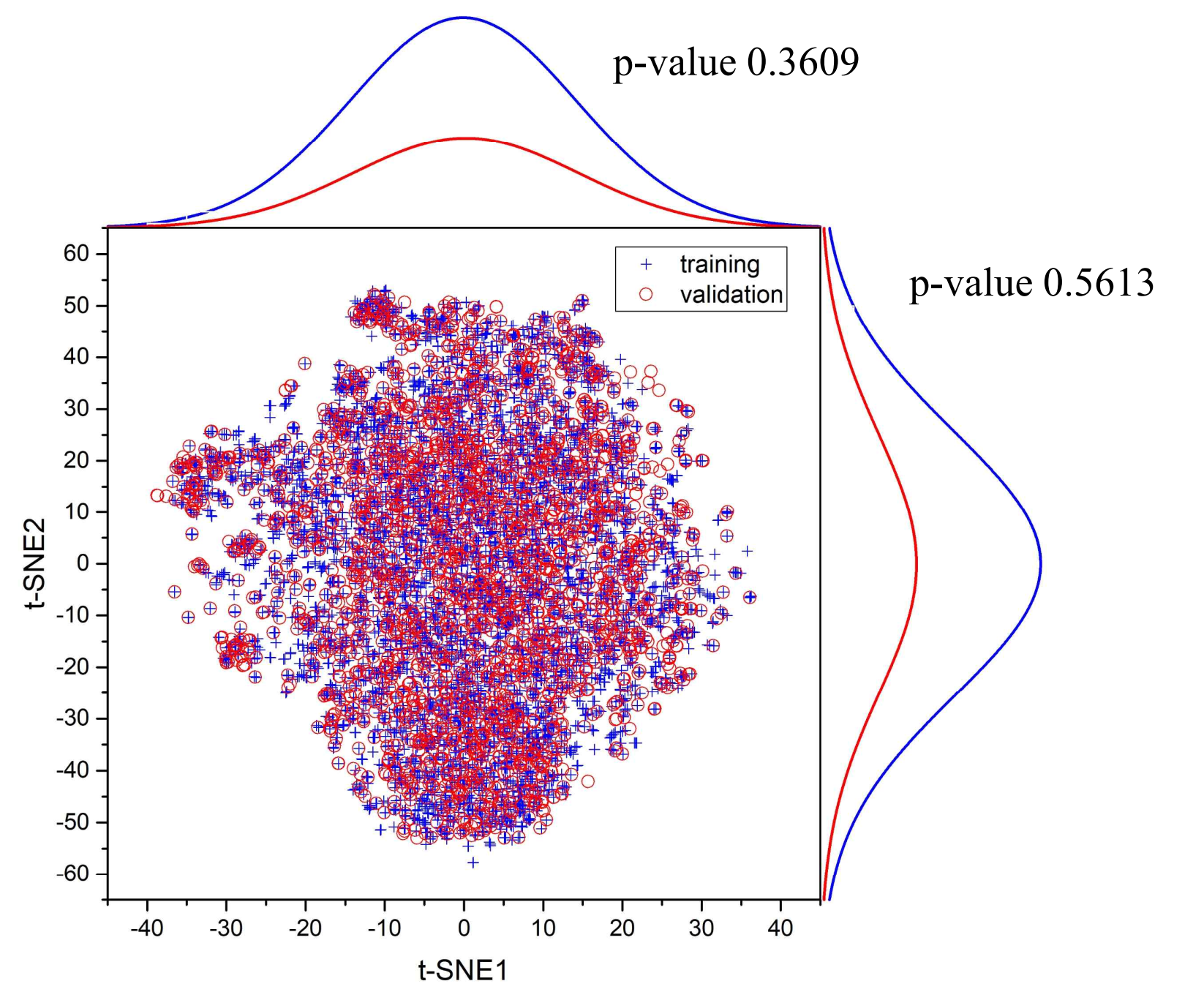


Supplementary Figure S2. 2D t-SNE map for training data and validation data
